# How does satisfaction of solar PV users enhance their trust in the power grid? - Evidence from PPAPs in rural China

**DOI:** 10.1186/s13705-021-00306-4

**Published:** 2021-09-16

**Authors:** Liping Ding, Yin Shi, Chenchen He, Qiyao Dai, Zumeng Zhang, Jiaxin Li, Ling Zhou

**Affiliations:** 1 School of Economics and Management, China University of Geosciences, No. 388 Lumo Road, Hongshan District, Wuhan, 430074 Hubei China; 2 Center for Energy and Environmental Management and Decision-making (CE2MD), China University of Geosciences, No. 388 Lumo Road, Hongshan District, Wuhan, 430074 Hubei China; 3 School of Foreign Languages, China University of Geosciences, No. 388 Lumo Road, Hongshan District, Wuhan, 430074 Hubei China

**Keywords:** Photovoltaic Poverty Alleviation Projects (PPAPs), Beneficiaries’ satisfaction, Trust in State Grid, Solar PV, Adoption

## Abstract

**Background:**

Photovoltaic Poverty Alleviation Projects (PPAPs) have been implemented in Chinese rural areas since 2014. As a new energy policy, PPAPs have played an important role in alleviating rural poverty. However, the adoption of solar PV faces multiple barriers from the perspective of beneficiaries. Therefore, this study aims to discuss and analyze factors affecting beneficiaries’ satisfaction and their trust in State Grid, promoting the adoption of solar PV.

**Methods:**

Based on the integrated American Customer Satisfaction Index (ACSI) and Unified Theory of Acception and Use of Technology (UTAUT) model, this study used the Structural Equation Model (SEM) to reveal how the beneficiaries’ satisfaction enhance their trust in State Grid. The data were obtained from a survey of 928 PPAPs’ beneficiaries by stratified and random sampling in Chinese rural areas.

**Results:**

The results confirm that environmental perception in this study has positive impact on beneficiaries’ satisfaction. In addition, perceived quality also has a positive effect on beneficiaries’ satisfaction and trust in State Grid; however, social influence has a negative impact on beneficiaries’ satisfaction; behavior expectation can directly promote beneficiaries’ satisfaction while indirectly propel their trust in State Grid.

**Conclusions:**

This study constructs an integrated customer satisfaction model from the perspective of beneficiaries and proposes relevant measures to promote the adoption of solar PV that can be applied to poverty reduction in other developing countries worldwide.

## Background

Nowadays, the global energy system has accelerated the transition to being low carbon. It has become an inevitable requirement to build a green-cycle and low-carbon energy system for social development, while solar energy and other renewable energies have shown huge potential and a great prospect. Since 2014, the Chinese government has been implementing the construction of Photovoltaic Poverty Alleviation Projects (PPAPs) which conform to the concept of green development, making great efforts to accelerate the speed of rural poverty alleviation [1]. From 2015 to 2017, National Energy Administration (NEA) has issued the special construction scales of PPAPs for 1.5 million kilowatts, 5.16 million kilowatts, and 4.19 million kilowatts, respectively, for three consecutive years [2]. As of 2020, the NEA has issued a total of 26.36 million kilowatts of PPAPs, benefiting nearly 60,000 poor villages and 4.15 million poor households, and providing 1.25 million public welfare jobs [3]. The national power industry statistics of 2020 released by NEA show that, although due to the impact of the COVID-19, the new installed capacity of PV power generation in China is still as high as 48.2 million kilowatts. By the end of 2020, the installed capacity of PV power generation in China is 253 million kilowatts, a year-on-year growth of 24.1% [4].

There are four types of PPAPs in China, mainly different in the scale of the power station, property rights, and income: (1) Household PV poverty alleviation power station (H-PVPA). The scale of this one is 3–5 kW, building on the roofs of poor households or in the open space of their courtyards. Besides, the property rights and income all belong to poor households. (2) Village-level PV poverty alleviation power station (V-PVPA). The power stations will be built on the land of village collectives with 100–300 kW installed capacity. Its property right belongs to the village collectives, and the income will be distributed proportionately between the village collectives and the poor households. (3) Greenhouse PV poverty alleviation power station (G-PVPA). Using modern agricultural facilities, such as agricultural greenhouses to build 1–100 MW PV power station. The property rights shall be owned jointly by investment enterprises and poor households. (4) Commercial PV poverty alleviation power station (C-PVPA). Large ground PV power stations with more than 10 MW installed capacity will be built on barren hills and slopes, while the property rights will be owned by the investment enterprises which will then donate part of the equity proceeds to the local government to be distributed to the poor households. The beneficiaries in this research mainly benefit from H-PVPA, V-PVPA, and C-PVPA.

However, as an innovative and targeted poverty reduction initiative, PPAPs must overcome current difficulties to achieve the expected results in a large scope. In previous researches, scholars have studied the internalities obstacles (quality of PV equipment, profit allocation mechanism, the institutional framework of energy policy management, etc.) and externalities obstacles (subsidy delays, environmental licensing challenges, etc.) to discuss the sustainable development of PPAPs [5–7]. With the rapid development of PPAPs, more and more rural residents participate in PPAPs as the main stakeholders, and their satisfaction (SS) is a key determinant for critical success factors (CSFs) in renewable energy projects (REPs) [8]. Therefore, their satisfaction should not be ignored for solar PV adoption. The State Grid is a monopolistic power supply corporation in China, whose technicians also play a major role in the progress of PPAPs. They need to proactively offer tracking services and develop a particular plan according to each customer to ensure the safe and stable operation of PV equipment. In the case of solar projects in Ghana, the continued growth of the solar market has been hampered by financing difficulties and the lack of local technicians and credit lines [9]. Only individuals who trust the installers and believe that the solar PV is beneficial are more likely to contact the installers and adopt solar PV [10].

Therefore, it is essential to reveal how the beneficiaries’ satisfaction enhances their trust in State Grid for PPAPs. It might promote the adoption of solar PV and contribute to poverty alleviation. To this end, this study constructs an integrated model adding the features of PPAPs and uses the AMOS software to explore the factors affecting beneficiaries’ satisfaction. In addition, it proposes relevant measures to promote the beneficiaries’ satisfaction with PPAPs, which may be useful for other developing countries’ poverty alleviation.

The rest of this paper is arranged as follows: the existing literature is reviewed and discussed in section ‘Theoretical foundation’. Section ‘Conceptual model and hypotheses’ will explain the conceptual model and propose the research hypotheses. The methodology and the results will be introduced, respectively, in sections ‘Methods and data’ and ‘Results’, followed by section ‘Discussion’, with the details of the impact of the results, theoretical contributions, and limitations. Finally, section ‘Conclusions and policy implications’ will conclude this paper with policy implications.

## Theoretical foundation

With the rapid development of satisfactory theories, scholars have adopted different theories and methods to study the satisfaction of their respective fields. In terms of the adopted theories, Gestalt theory [11], Grounded theory [12], Three-factor theory of customer satisfaction [13], and Satisfaction spillover theory [14] are widely applied to explore satisfaction in various fields. The most common theory is the Customer Satisfaction Index (CSI). In 1989, CSI was originally established by Sweden [15], namely, the Swedish Customer Satisfaction Barometer Index (SCSBI). Based on this, a new factor “perceived quality” was added to establish an American Customer Satisfaction Index (ACSI) model [16]. At present, scholars also begin to adopt the extended Unified Theory of Acceptance and Use of Technology 2 (UTAUT2) to study customer satisfaction of mobile food ordering or mobile commerce [17]. UTAUT2 added three new factors of “hedonic motivation”, “price value” and “habits” on the basis of Unified Theory of Acceptance and Use of Technology (UTAUT) [18]. UTAUT mainly explored the impact of four variables: “performance expectancy”, “effort expectancy”, “social influence”, and “facilitating conditions” on users’ willingness to use behavior [19]. In terms of the adopted methods, qualitative methods such as the fuzzy analytic hierarchy process [20] and the evaluation method based on rough set conditional information entropy [21] are adopted to establish the attribute weight of satisfaction. Furthermore, some scholars adopted quantitative methods such as the cross-domain hybrid method [22] and partial least squares method based on SEM technology [23, 24] to evaluate satisfaction.

SEM or path analysis is mostly used in satisfaction studies to explore the causal relationship among variables [25]. In the field of renewable energy, the important factors determining consumers’ satisfaction included the image of service provider, consumer expectation, and perceived quality, etc. [24]. Specifically, in the research of solar PV, the benefits of the solar household system (SHS) lifestyle and the quality of its equipment played a key role in improving users’ satisfaction with SHS in rural Bangladesh from a quantitative perspective [26, 27]. At the same time, public satisfaction played a positive role in using solar technology [28]. Information and educational campaigns about clean energy technology might have a positive impact on homeowners’ satisfaction, leading to positive word-of-mouth recommendations and other impacts [26]. In a survey of distributed solar technology adoption in rural India, it was found that the use of home solar technology is closely related to the subjective satisfaction of home lighting [29]. In addition, scholars also analyzed other factors affecting satisfaction, such as public trust [30], purchase intention [31], and government image [32].

Trust is regarded as a critical feature and a central mechanism in business transactions [33]. In the expansion of modern coal-fired power plant projects and power grid projects, trust exerted a significant influence on public support attitudes [34, 35]. For environmentally sustainable development, companies need to invest resources to increase customers’ green perceived value, thereby enhancing green trust and customers’ green loyalty [36]. Previous pieces of literature have shown there is a direct and indirect correlation between satisfaction and trust. For the former, some scholars found that the satisfaction of neighborhood facilities was an important predictor of social trust [37]. Otherwise, the trust in local government also had a significant positive impact on urban residents’ environmental public service satisfaction, while the trust in central government had no significant impact [38]. Thus, it was necessary to improve residents’ trust in local governments with communication and cooperation. For the latter, trust played a certain intermediary or mediation role when scholars discussed the relationship among satisfaction of service quality [39], organizational culture and leadership performance [40], and manufacturer–supplier [41]. Accordingly, some scholars also used satisfaction as an intermediary variable to discuss the influence between trust with green perceived quality, green perceived risk [42], and relationship benefits [43].

To sum up, this study will use the integrated model of ACSI and UTAUT to explore the factors affecting satisfaction. At present, most researches explore the influence of users’ satisfaction and their behavior based on a single model, but few types of research combine two or more models. What’s more, most scholars usually take “loyalty” or “complaint” as the outcome variable of satisfaction to explore the relationship between them. There are few scholars to discuss the direct relationship between satisfaction and trust. Therefore, this study will add the perceived variable of “environmental perception”, and take “trust in the State Grid” as the behavioral outcome variable for beneficiaries’ satisfaction.

## Conceptual model and hypotheses

**Fig. 1:**
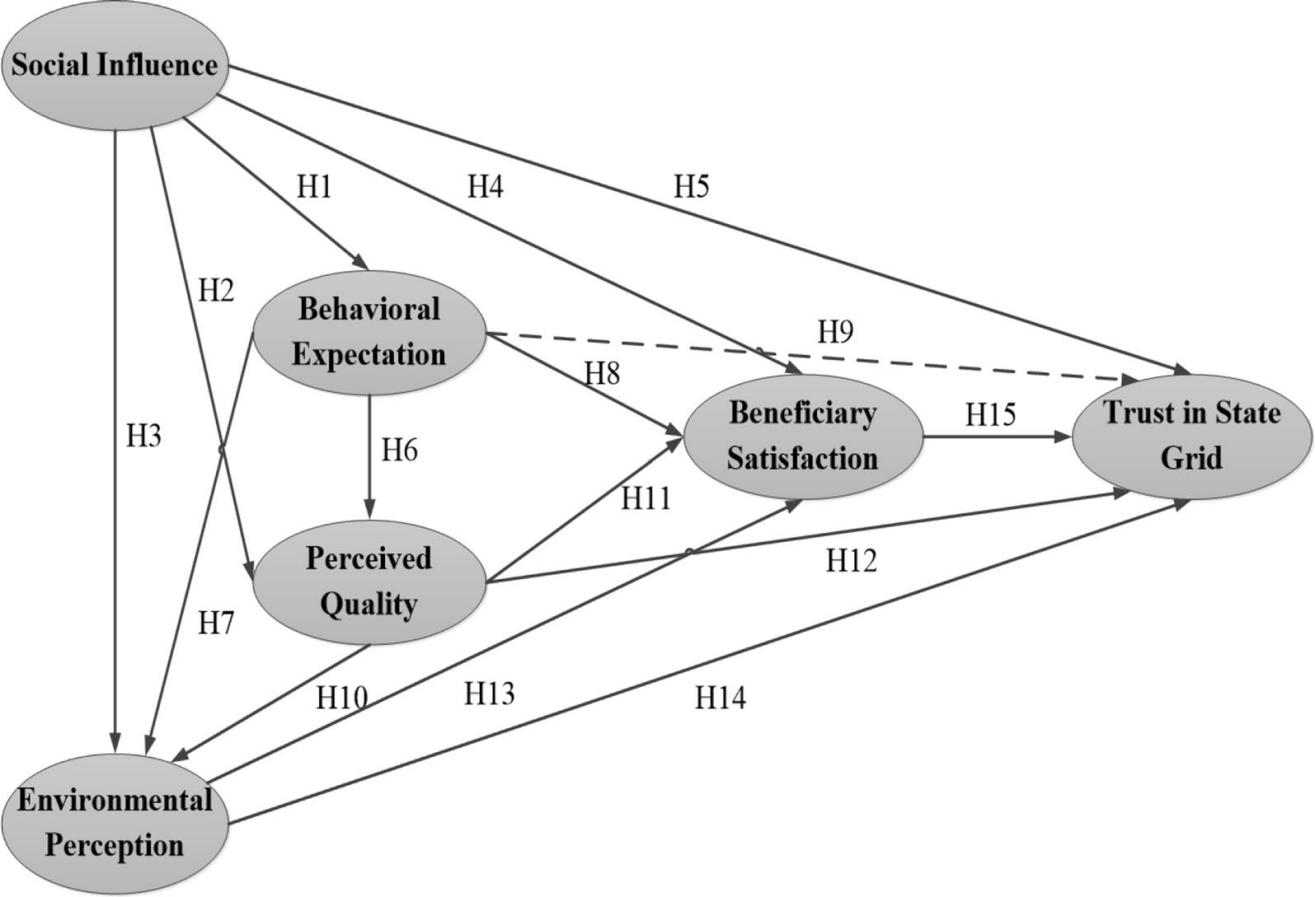
Conceptual model of this study

Based on the ACSI model, this study builds beneficiaries’ satisfaction index of PPAPs. Among them, perceived value is a subjective feeling of customers on their benefits after integrating quality and price [44], while PPAPs generally involve the investment of three major bodies, namely, the State Grid, the government, and enterprises. Thus, the beneficiaries do not need to afford the high investment. The total incomes of the PPAPs are directly shared by the beneficiaries or together with the State Grid and village collectives. Therefore, this study will not consider the influencing factors of perceived value. The complaint and loyalty, respectively, represented the degree of users’ dissatisfaction and lack of trust in the service provided by the product [45]. This study will combine these two consequence variables into one to explore the beneficiaries’ trust in State Grid, which includes both the beneficiaries’ judgment on the PPAPs’ services provided by the State Grid and the beneficiaries’ credibility on it. At the same time, “environmental perception” will be added to the original model to further explore the factors influencing the satisfaction of PPAPs. Figure 1 shows the proposed research framework of this study.

### Social influence

Social influence means that the extent to which an individual perceives it significant, while others believe they should apply the new system [46]. Solar energy is emerging renewable energy, the beneficiaries are not fully familiar with it. Their satisfaction with PPAPs is primarily influenced by the views, ideas, and attitudes of others. Thus, this study defines social influence as the extent to which the beneficiaries perceive the government officials and village leaders who persuade them to adopt solar PV. The influence of government officials or village leaders is also an external environmental impact. It was found that the external environment had an indirect influence on the outcome expectation in exploring the influencing factors of farmers’ participation in the joint-stock cooperative system [47]. Therefore, this study proposes the following hypothetical path:

#### H1:

Social influence will have a positive impact on the behavioral expectation with PPAPs.

Social interaction can be divided into “social interaction with employees” and “social interaction with peers”, and both of which had a significant positive impact on perceived service quality [48]. For this study, government officials and village leaders play a key role in raising beneficiaries’ awareness of the benefits of solar energy or the project in their daily interactions. Therefore, this study proposes the following hypothetical path:

#### H2:

Social influence will have a positive impact on the perceived quality with PPAPs.

Environmental perception is an element of judging the government’s environmental governance. The more serious the public perceives environmental pollution, the lower the local government’s evaluation will be. Consumers’ cognition and preference for ecological technology were affected by society. Thus, it would underestimate the potential of consumers’ choice for the emerging environmental technologies when neglecting the process of social influence [49]. Therefore, this study proposes the following hypothetical path:

#### H3:

Social influence will have a negative impact on environmental perception with PPAPs.

It was confirmed that social influence affected citizen satisfaction through developing a model for compelling citizens to adopt e-government technology [50]. Customers could gain social acceptance from others when using a product, which would simultaneously increase social value and satisfaction [51]. Therefore, this study proposes the following hypothetical path:

#### H4:

Social influence will have a negative impact on beneficiaries’ satisfaction with PPAPs.

Trust was an essential driving force for consumers’ shopping decisions in social commerce [34]. Consumers developed familiarity and trust in the products when engaging in social interactions [52]. Personal factors, community factors, and social factors all significantly affected residents’ social trust, which demonstrated that the improvement of society trust not only need individual efforts but also needs intermediary organizations’ progress [53]. Therefore, this study proposes the following hypothetical path:

#### H5:

Social influence will have a positive impact on trust in State Grid with PPAPs.

### Behavioral expectation

Expectation represents both prior consumption experience with its offering and a forecast of the company’s ability to provide quality in the future [16]. The government is involved to ensure the quality of implementing PPAPs. Therefore, this study defines behavioral expectation as the degree to which the beneficiaries expected the government’s behavior with PPAPs. In the energy sector, it was found that consumer expectation has a positive relationship with perceived quality [24]. Otherwise, it was found that public expectation had a positive impact on perceived quality in the study of building a service-oriented government [54]. Therefore, this study proposes the following hypothetical path:

#### H6:

Behavioral expectation will have a positive impact on perceived quality with PPAPs.

A lot of researches showed that users’ expectation and environmental perception had direct or indirect effects on users’ satisfaction and loyalty [55], but the relationship between them still has not been discussed. At present, PPAPs are still in a period of continuous development, and they still require subsidies and support from the government. Only when government attaches great importance can farmers better understand the significance. Therefore, this study proposes the following hypothetical path:

#### H7:

Behavioral expectation will have a positive impact on environmental perception with PPAPs.

Public satisfaction could be jointly influenced by three variables: public expectation, perception of public service quality, and perceived difference in service effectiveness [56]. Other researches had also confirmed that passenger’s expectation was positively correlated with passengers’ perceived quality and their satisfaction [54, 57]. Therefore, this study proposes the following hypothetical path:

#### H8:

Behavioral expectations will have a positive impact on beneficiaries’ satisfaction with PPAPs.

Previous studies have explored the effects of customer expectation (antecedent variable) and perceived trust (outcome variable) on satisfaction, with perceived trust acting as a mediator. In the area of the public to use E-government, it was found that effort expectation had a significant impact on perceived trust as an internal belief factor [58]. Therefore, this paper proposes the following hypothetical path:

#### H9:

Behavior expectation will have a positive impact on trust in State Grid with PPAPs.

### Perceived quality

Perceived quality is the service quality that customers perceived, while the concept of service quality is defined as a comparison between expectation and actual service performance [59]. In this paper, perceived quality is defined as the beneficiaries’ perceive quality changes in family energy use and environmental problems after the adoption of solar PV. Scholars found that there was a positive relationship between perceived quality and consumers’ awareness of environmental protection [60]. Consumers will feel a high quality about the product if it has an environmental label on the package, which will further enhance their environmental awareness [61]. Therefore, this study proposes the following hypothetical path:

#### H10:

Perceived quality will have a positive impact on environmental perception with PPAPs.

Perceived service quality can be divided into three dimensions, including platform perceived service quality, bicycle entity perceived quality, and value perceived quality. The platform and bicycle entity perceived service quality was found to significantly affect users’ satisfaction [62]. In addition, the equipment quality of solar home systems (SHS) played an essential role in improving users’ satisfaction in rural areas [27]. Therefore, this study proposes the following hypothetical path:

#### H11:

Perceived quality will have a positive impact on beneficiaries’ satisfaction with PPAPs.

An indirect relationship between e-service quality and green trust was found to explore the factors influencing green purchase intention [63]. However, other researchers found that perceived quality had a direct and positive impact on trust, namely, green perceived quality positively affected green trust and the relationship between them was partially moderated by green satisfaction [36]. Therefore, this study proposes the following hypothetical path:

#### H12:

Perceived quality will have a positive impact on trust in State Grid with PPAPs.

### Environmental perception

Environmental perception can be divided into two aspects. One refers to the image formed by the environment in an individual’s mind. The other refers to the feeling that the quality of the environment brings to the individual [64]. The environmental perception in this paper refers to the beneficiaries’ perception of environmental quality due to excessive use of non-renewable energy. The environmental performance had a significant positive impact on customers’ satisfaction [65]. Comparing the non-electric vehicles (EV) users’ purchase intention with the post-purchase satisfaction, environmental perception had a direct impact on the purchase intention of non-EV users, whereas it had an indirect impact on the post-purchase satisfaction of EV users [66]. Therefore, this study proposes the following hypothetical path:

#### H13:

Environmental perception will have a positive impact on beneficiaries’ satisfaction with PPAPs.

The perception of environmental problems was an essential factor in low-carbon behavior [63], while social trust had a moderating effect between environmental fairness perception and farmers’ low carbon production behavior [67]. In addition, environmental impact assessment (EIA) tended to increase public trust, the perceived fairness and reduce the perceived risk in the global site selection of waste incineration facilities [68]. Therefore, this study proposes the following hypothetical path:

#### H14:

Environmental perception will have a positive impact on trust in State Grid with PPAPs.

### Beneficiaries’ satisfaction

Satisfaction is an overall affective response to a perceived discrepancy between prior expectation and perceived performance after consumption [69]. This study defines satisfaction as the sense of happiness formed by the beneficiaries through the previous expectation and actual perception. Some studies confirmed there was a close connection between trust and users’ satisfaction in mobile commerce [70, 71]. In the research on users’ word-of-mouth intentions of the green hotel industry, consumers’ green satisfaction also had a significant effect on green trust [72]. Therefore, this study proposes the following hypothetical path:

#### H15:

Beneficiaries’ satisfaction will have a positive impact on trust in State Grid with PPAPs.

## Methods and data

### Data collection and participants

**Fig. 2:**
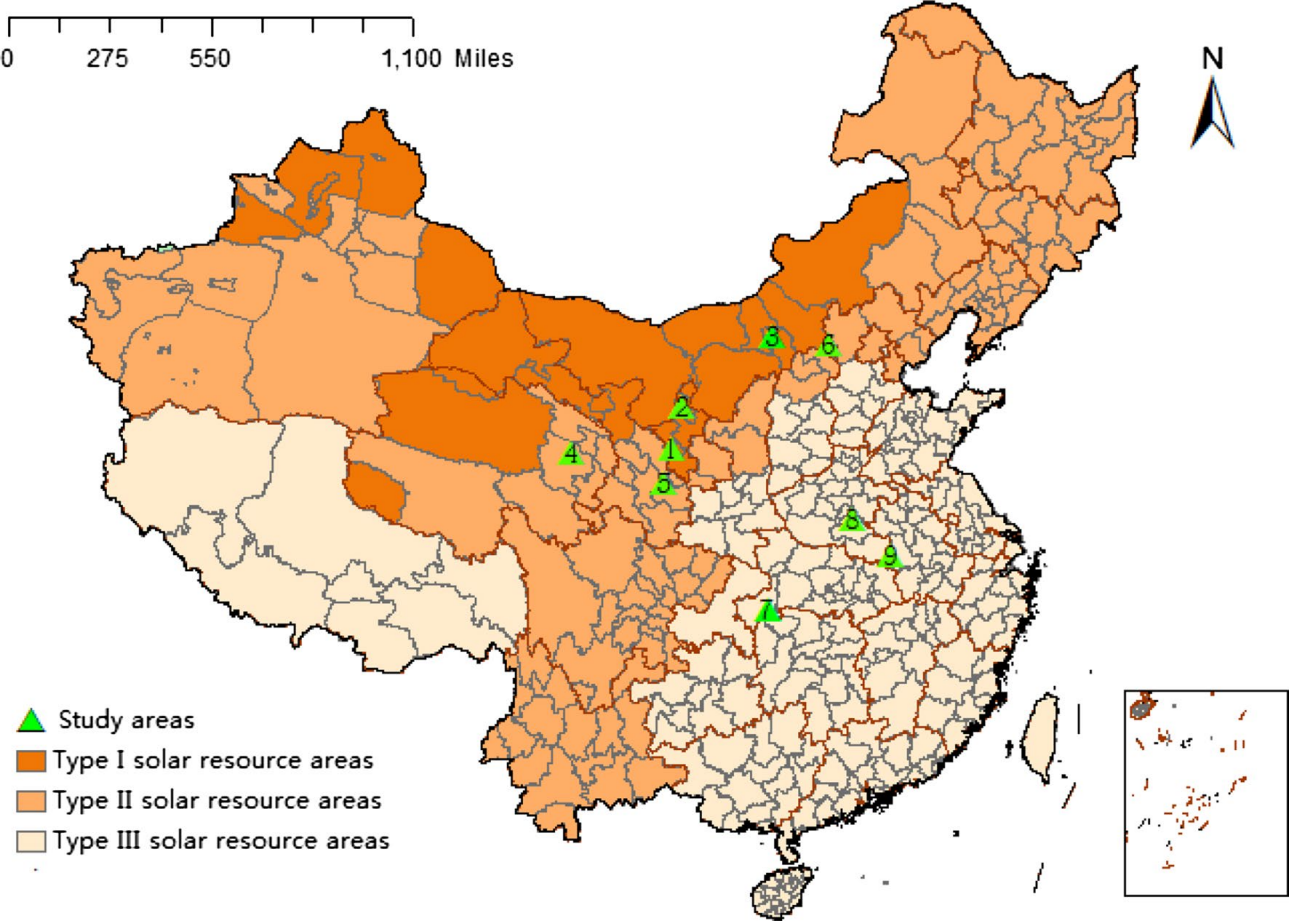
Distribution of study areas. *Notes* 1—Haiyuan county; 2—Yongning county; 3—Chahar Right Middle Banner; 4—Gonghe county; 5—Tongwei county; 6—Tianzhen county; 7—Changyang county; 8—Shangcai county; 9—Jinzhai county

This empirical study was an extensive sample questionnaire conducted in eight provinces in China. The surveyed areas were selected considering different sunlight levels, which were divided into, three types of solar energy resource areas by the National Development and Reform Commission (NDRC). Therefore, this study selected three sample counties from each type with a total of nine sample counties selected. That is, Type I solar resource areas (≥ 1600 h): Yongning in Ningxia, Haiyuan in Ningxia and Chahar Right Middle Banner in Inner Mongolia; Type II solar resource areas (1400–1600 h): Gonghe in Qinghai, Tingwei in Gansu, and Tianzhen in Shanxi; Type III solar resource areas (1200–1400 h): Changyang in Hubei, Shangcai in Henan and Jinzhai in Anhui (see Fig. 2). As for the selection of village-level samples, considering that the number of PPAPs implemented in each village was different, we carried out village-level sampling by the principle of selecting more than 50% poor villages in each county with a total of 36 project villages were selected. Finally, we randomly selected from the villagers’ list ensuring that 20–30 poor households were selected from each project village (see Fig. 4 in Appendix). From June 2018 to September 2018, the research group selected survey samples based on the principles of stratified and random sampling and conducted a face-to-face interview with beneficiaries in the field survey.

### Questionnaire

The main contents of the questionnaire include the basic characteristics of the household population, such as gender, age, household register, education background, whether they are village leaders or not. Otherwise, it also includes what kind of PPAPs they have benefited from, the satisfaction degree with the implementation of PPAPs, the perception of environmental conditions, energy use situation, etc. The alpha value of Cronbach was also tested. According to the recommendations of Nunnally [73], all construction values should be higher than 0.70.

As shown in Table 1, the total number of respondents in this study was 940, and 928 (98.7%) valid questionnaires were obtained after deleting incomplete and inconsistent questionnaires. The object of this study is rural residents who benefited from PPAPs. The majority (76.9%) of beneficiaries interviewed were male. The beneficiaries within the age group of 50–59 years were about 27.6%, while beneficiaries of 40–49 years and 60–69 years, respectively, accounted for 23.5% and 23.2%, and the age group less than 20 years (0.6%) was the smallest proportion. In terms of education level, the largest group was primary school degrees (50.4%), followed by high school degrees (26.4%). Regarding household registration, most of the current samples (98.6%) were those who have rural household registration, about 1.4% were non-farm household registration. Among the beneficiaries interviewed, most (98.3%) belong to ordinary villagers, while only 1.7% belong to village leaders.

**Table 1:** Demographic characteristics of beneficiaries

Demographic profile	Number of beneficiaries (*N* = 928)	Percentage (%)
Gender		
Male	714	76.9
Female	214	23.1
Total	928	100
Age		
< 20	6	0.6
20–29	24	2.6
30–39	62	6.7
40–49	218	23.5
50–59	256	27.6
60–69	215	23.2
> 69	147	15.8
Total	928	100.0
Education level		
Illiteracy	165	17.8
Primary school	468	50.4
Junior high school	245	26.4
High school	45	4.8
Specialist	2	0.2
University	3	0.3
Total	928	100.0
Household registration		
Rural area	915	98.6
Town	13	1.4
Total	928	100.0
Whether a village leader		
Yes	912	98.3
No	16	1.7
Total	928	100.0

Source: The authors compiled the data through the questionaire survey, which conducted a 3-month field survey from June, 2018 to September, 2018

## Results

Preliminary data analysis and test of statistics will be first provided in section ‘Descriptive statistics’. Then the two-stage SEM will be introduced, which was used to verify the conceptual model and test its associated hypotheses. The results of confirmatory factor analysis (CFA) in the first phase are provided in section ‘Confirmatory factor analysis’, and the structural model of the SEM in the second stage is presented in section ‘Structural model’.

### Descriptive statistics

As shown in Table 2, beneficiaries seem to have a relatively satisfactory view of PPAP for all the aspects considered in the current study. For example, the average mean of behavioral expectation items was 4.578, while the average standard deviation was 0.672, which indicated that the beneficiaries were positive about the government’s behavior in PPAPs. The quality improvement brought by PPAPs was relatively obvious for the beneficiaries, as the average mean of perceived quality items was 3.469 (0.933). Likewise, the implementation of PPAPs was considered satisfactory for the beneficiaries, since the average mean of beneficiaries’ satisfaction items was 3.920 (0.643). The majority of participants believed that the State Grid was trustworthy in PPAPs, as the average mean of trust in State Grid items was 3.997 (0.786). In addition, the average mean of these two factors was as follows: social influence (3.506; 1.168) and environmental perception (3.504; 0.870), which means that the beneficiaries also gave positive evaluations for the two innovative factors added to the PPAPs.

**Table 2:** Descriptive statistics of the scale items (mean and standard deviation)

Constructs	Item	Mean	Standard deviation
Social influence	SI 1	3.412	1.2008
	SI 2	3.533	1.1505
	SI 3	3.574	1.1532
	**Average**	**3.506**	**1.1682**
Behavioral expectation	BE 1	4.558	0.6690
	BE 2	4.568	0.7483
	BE 3	4.489	0.6935
	BE 4	4.697	0.5782
	**Average**	**4.578**	**0.6724**
Perceived quality	PQ 1	3.319	0.8069
	PQ 2	3.366	0.9413
	PQ 3	3.723	1.0515
	**Average**	**3.469**	**0.9332**
Environmental perception	EP 1	3.575	0.8340
	EP 2	3.422	0.8779
	EP 3	3.515	0.8982
	**Average**	**3.504**	**0.8700**
Beneficiaries’ satisfaction	BS1	3.860	0.6649
	BS 2	3.871	0.6606
	BS 3	3.843	0.6428
	BS 4	3.955	0.6454
	BS 5	4.072	0.5968
	**Average**	**3.920**	**0.6432**
Trust in State Grid	TSG 1	4.000	0.7461
	TSG 2	4.011	0.7532
	TSG3	3.920	0.8591
	**Average**	**3.9770**	**0.7861**

Source: From the calculation results of SPSS 22.0 and AMOS 23.0 software by the authors

The bold indicates the average value of every construct

Based on the mature scale and measurement items exploring adoption willingness designed by scholars, this study designed the Likert five-point scale questionnaire [74, 75]. As shown in Table 3, there are 6 latent variables and 21 items in the questionnaire. SI represents social influence, BE refers to behavioral expectation, PQ is perceived quality, and EP denotes environmental perception, BS represents beneficiaries’ satisfaction, TSG stands for trust in State Grid. In all measured variables, the kurtosis coefficient (kurtosis) is less than 8, and the skew coefficient (skew) is less than 3. It can be considered that the data conform with the normal distribution in general.

**Table 3:** Topic design of latent variables

Latent variable	Item	Kurtosis	Skew
Social Influence (SI)	SI1 Government officials want me to use solar PV power generation	− 0.622	− 0.502
	SI 2 The poverty alleviation leader in the village hope that I will use solar PV power generation	− 0.248	− 0.660
	SI 3 Village leader want me to use solar PV power generation	− 0.315	− 0.610
Behavioral Expectation(BE)	BE 1 I hope the government will honor its promise and give us the subsidies we deserve	2.656	− 1.587
	BE 2 I hope the government will strengthen the maintenance of solar PV power generation facilities	1.984	− 1.435
	BE 3 I hope that the solar PV policy will remain stable and not become too fast	1.256	− 1.272
	BE 4 I hope the government can provide us with all the support needed for solar PV power generation projects	4.738	− 2.064
Perceived Quality (PQ)	PQ 1 I will be able to better manage household energy use	− 0.108	0.136
	PQ 2 I will be able to better control household energy expenditure	− 0.258	− 0.104
	PQ 3 Our community/village will be able to better protect the environment	− 0.252	− 0.562
Environmental Perception (EP)	EC 1 I am concerned about environmental problems such as air and water pollution caused by excessive use of energy	− 0.348	− 0.067
	EC 2 I’m worried that excessive use of energy will increase carbon emissions	− 0.133	0.065
	EC 3 I worry that excessive use of energy will cause the natural environment to be unable to recover	− 0.243	− 0.054
Beneficiaries’ Satisfaction (BS)	BS 1 How do you think the rationality of collective income distribution of PPAPs	0.895	− 0.541
	BS 2 How satisfied are you with the subsidy for PPAPs	0.274	− 0.304
	BS 3 How satisfied are you with the follow-up management and protection of PPAPs	1.426	− 0.600
	BS 4 How satisfied are you with the implementation of PPAPs	1.463	− 0.584
	BS 5 How do you think the sustainability of PPAPs	− 0.224	− 0.025
Trust in State Grid (TSG)	TSG 1 I believe that State Grid is credible in PPAPs	0.346	− 0.483
	TSG 2 I believe that State Grid provides good service in PPAPs	0.609	− 0.578
	TSG 3 I believe that State Grid has relations with their customers	1.055	− 0.827

Source: these questions are designed by the authors based on the previous literature

### Confirmatory factor analysis

From the KMO and the Bartlett sphericity test, the KMO value was 0.800, indicating that the sample data had high validity. The significance level of the Bartlett sphericity test was 0.000 less than 0.005. Therefore, the null hypothesis of the Bartlett sphericity test was rejected and the data was considered suitable for factor analysis. The principal component analysis method was adopted to perform exploratory analysis with the data, and five common factors were set to be extracted, and then the maximum variance method was used to rotate the factor. The factor load matrix after the rotation is shown in Table 4, the factor load values of each measurement item on its associated variable were all greater than 0.50, and the factor load of the cross-measure item did not exceed 0.50, indicating that the scale had good convergence and discriminant validity.

**Table 4:** Factor loading matrix by orthogonal method

Variable	SI	BE	PQ	EP	BS	TSG
SI 1	0.915					
SI 2	0.916					
SI 3	0.917					
BE 1		0.783				
BE 2		0.821				
BE 3		0.792				
BE 4		0.757				
PQ 1			0.863			
PQ 2			0.879			
PQ 3			0.734			
EP 1				0.844		
EP 2				0.830		
EP 3				0.868		
BS 1					0.805	
BS 2					0.841	
BS 3					0.729	
BS 4					0.797	
BS 5					0.684	
TSG 1						0.775
TSG 2						0.860
TSG 3						0.844

Source: The results based on SPSS 22.0 software calculations by the authors

The reliability and validity of the measurement model were further analyzed using multiple criteria. First, Cronbach’s alpha and composite reliability (CR) was adopted to test the internal consistency of the variables. CR values for all latent variables were calculated and found to be not less than 0.70 [76, 77]. As shown in Table 5, the largest value of CR was for SI (0.9438), whereas the smallest value of CR was recorded for TSG (0.8118). Likewise, the Cronbach’s alpha values of all latent variables were higher than their critical values of 0.70. SI had the largest Cronbach’s alpha value (0.944), while the lowest value of Cronbach’s alpha was for TSG (0.805), indicating that the model had high reliability.

**Table 5:** Construct validity and reliability

Variable	Cronbach’s alpha	CR	AVE
SI	0.944	0.9438	0.8484
BE	0.814	0.8167	0.5277
PQ	0.821	0.8440	0.6484
EP	0.845	0.8239	0.6171
BS	0.840	0.8201	0.4800
TSG	0.805	0.8118	0.5913

Source: The results based on the AMOS 23.0 software calculations by the authors

The factor loading value (Estimate) of each latent variable corresponding to the observed variable was considered to test the convergence validity [77]. It was generally required that factor loading value and average variance extracted (AVE) were greater than 0.50. The factor loading values in Table 6 were all greater than 0.5, which were in line with the recommendations of [77]. The model is a very ideal state when the factor loading value was greater than 0.71 and the AVE value was 0.50. Accordingly, it is good when the factor loading value was greater than 0.63, and the AVE value was 0.40 [78]. AVE values corresponding to each latent variable in Table 5 were greater than 0.5, indicating that these variables were in a very ideal state. Though the AVE value of beneficiaries’ satisfaction was 0.480, it still indicated that the variable was in a good condition. Therefore, the above shows that the model had good convergence validity. The discriminant validity of latent variables was also tested. If the correlation coefficients were less than the square roots of their corresponding AVE values, then it can be considered that different variables have obvious discriminant validity [76]. As shown in Table 7, the correlation coefficient of each variable was less than the square root of its corresponding AVE value, so the model is considered to have good discriminant validity.

**Table 6:** Standardized regression weights (factor loading)

Items		Latent construct	Estimate
SI 1	< —	SI	0.907
SI 2	< —	SI	0.938
SI 3	< —	SI	0.918
BE 1	< —	BE	0.764
BE 2	< —	BE	0.751
BE 3	< —	BE	0.730
BE 4	< —	BE	0.656
PQ 1	< —	PQ	0.867
PQ 2	< —	PQ	0.894
PQ 3	< —	PQ	0.628
EP 1	< —	EP	0.666
EP 2	< —	EP	0.973
EP 3	< —	EP	0.679
BS 1	< —	BS	0.598
BS 2	< —	BS	0.654
BS 3	< —	BS	0.687
BS 4	< —	BS	0.828
BS 5	< —	BS	0.676
TSG 1	< —	TSG	0.686
TSG 2	< —	TSG	0.828
TSG 3	< —	TSG	0.786

Source: The results based on the AMOS 23.0 software calculations by the authors

**Table 7:** Discriminant validity

Variable	SI	BE	PQ	EP	BS	TSG
SI	0.921					
BE	0.264	0.726				
PQ	0.308	0.143	0.805			
EP	− 0.34	0.245	0.28	0.786		
BS	− 0.17	0.108	0.085	0.225	0.693	
TSG	0.08	0.029	0.182	0.205	0.182	0.769

Source: The results based on the AMOS 23.0 software calculations by the authors

Furthermore, CFA was involved to test the applicability of the model at the first phase. The models' overall fit evaluation indexes were considered, such as absolute adaptation indexes (*χ*^2^/*df*, RMR, SRMR, RMSEA, GFI, and AGFI), value-added adaptation indexes (NFI, RFI, IFI, TLI, and CFI), and simple adaptation indexes (PGFI, PNFI, CN, and PCFI). As shown in Table 8, one index of absolute adaptation indexes (SRMR) was not within the standard level, and SRMR was close to the adaptation standard. To sum up, the theoretical model constructed in this study has a good fit for the sample data.

**Table 8:** Fit indexes

Fit indexes	Recommended value	Measurement model
Absolute Fit Indexes		
*χ*^2^/*df*	≤ 5	4.156
RMR	≤ 0.05	0.046
SRMR	≤ 0.05	0.0545
RMSEA	≤ 0.08	0.058
GFI	≥ 0.9	0.928
AGFI	≥ 0.9	0.903
Value-Added Fitness Indexes		
NFI	≥ 0.9	0.931
RFI	≥ 0.9	0.916
IFI	≥ 0.9	0.947
TLI	≥ 0.9	0.935
CFI	≥ 0.9	0.947
Minimal Fit Indexes		
PGFI	≥ 0.5	0.691
PNFI	≥ 0.5	0.763
CN	≥ 200	283
PCFI	≥ 0.5	0.775

Source: The results based on the AMOS 23.0 software calculations by the authors

Finally, a common method deviation test was conducted by considering Harman’s single factor [59, 79]. Harman’s single factor test for EFA was conducted on 21 observed variables and was checked with a non-rotation factor solution. It was found that there were no newly recorded factors, and the variation rate of the first factor was recorded as 21.401%. According to the suggestion of Podsakoff [59], this value was not higher than 50%. Thus, the deviation test can be used for the current research data.

### Structural model

**Fig. 3:**
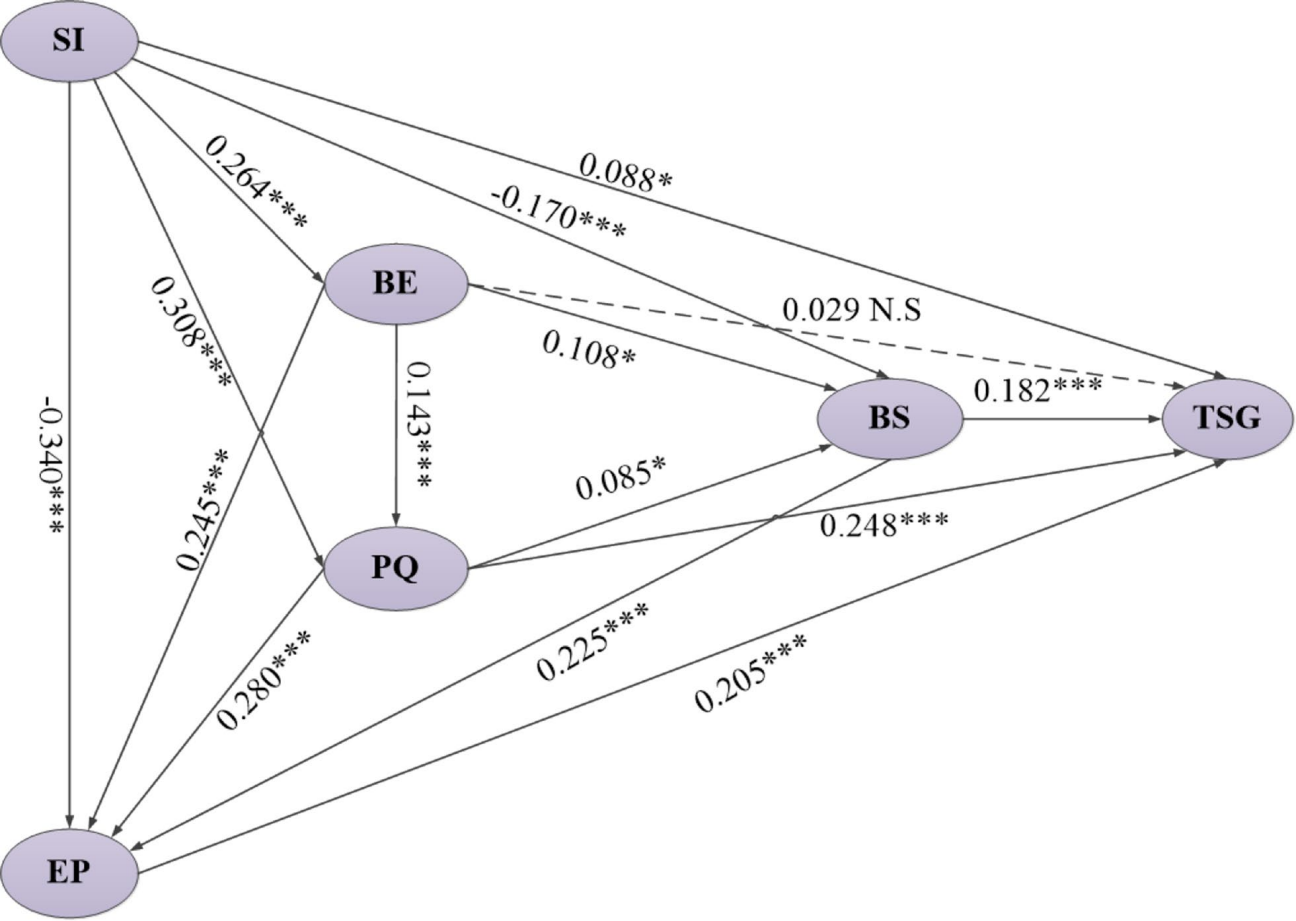
Validation of the conceptual model. *Notes* *p < 0.05; **p < 0.01; ***p < 0.001

**Fig. 4:**
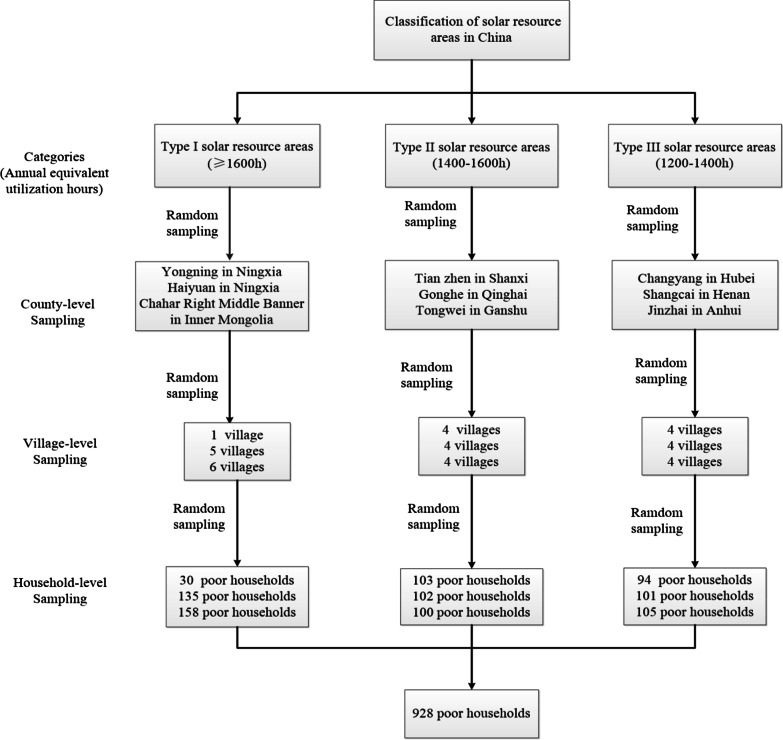
Sample size of study areas. *Notes* The selecting principle in village-level sampling: more than 50% project villages were selected in each sample county; The selecting principle in household-level sampling: 20–30 poor households were selected from each project village

In the second phase, AMOS 23.0 was used to test the research hypotheses of the conceptual model. The conceptual model also supported prediction validity. As for the test of the research hypotheses (Table 9), the results of the path coefficient analysis showed that beneficiaries’ satisfaction was significantly affected by the role of SI (*γ* = − 0.170, *p* < 0.001); BE (*γ* = 0.108, *p* < 0.05); PQ (*γ* = 0.085, *p* < 0.05); EP (*γ* = 0.225, *p* < 0.001). As for the main causal path leading to grid corporation trust, the results supported the significant effect of SI (*γ* = 0.088, *p* < 0.05); PQ (*γ* = 0.248, *p* < 0.001); EP (*γ* = 0.205, *p* < 0.001); BS (*γ* = 0.182, *p* < 0.001). Although BE (*γ* = 0.029, *p* > 0.05) didn’t directly affect the beneficiaries’ trust in State Grid, it can be indirectly affected by perceived quality. In addition, the results also confirmed that there was an interaction among these factors. For example, BE (*γ* = 0.264, *p* < 0.001); PQ (*γ* = 0.308, *p* < 0.001); EP (*γ* = − 0.0.340, *p* < 0.001) will be affected by social influence, while PQ (*γ* = 0.143, *p* < 0.001); EP (*γ* = 0.245, *p* < 0.001) will be affected by behavioral expectation, and perceived quality will also affect EP (*γ* = 0.245, *p* < 0.001), as shown in Fig. 3.

**Table 9:** Results of hypotheses testing

Research hypotheses	Hypothesized path	Unstandardized path coefficient estimation	S.E.	C.R.	*p*	Standardized path coefficient estimation	Accept/Reject	VIF
H1	BE < — SI	0.124	0.017	7.093	***	0.264	Accept	1.000
H2	PQ < — SI	0.198	0.023	8.508	***	0.308	Accept	1.056
H3	EP < — SI	− 0.173	0.022	− 8.047	***	− 0.340	Accept	1.196
H4	BS < — SI	− 0.062	0.016	− 3.944	***	− 0.170	Accept	1.256
H5	TSG < — SI	0.041	0.020	2.116	0.034	0.088	Accept	1.288
H6	PQ < — BE	0.195	0.052	3.722	***	0.143	Accept	1.056
H7	EP < — BE	0.267	0.044	6.038	***	0.245	Accept	1.086
H8	BS < — BE	0.084	0.033	2.545	0.011	0.108	Accept	1.131
H9	TSG < — BE	0.029	0.041	0.703	0.482	0.029	Reject	1.136
H10	EP < — PQ	0.223	0.033	6.823	***	0.280	Accept	1.200
H11	BS < — PQ	0.048	0.024	1.998	0.046	0.085	Accept	1.269
H12	TSG < — PQ	0.182	0.031	5.893	***	0.248	Accept	1.277
H13	BS < — EP	0.161	0.030	5.344	***	0.225	Accept	1.128
H14	TSG < — EP	0.189	0.038	5.020	***	0.205	Accept	1.157
H15	TSG < — BS	0.234	0.053	4.427	***	0.182	Accept	1.075

Source: The results based on the AMOS 23.0 software calculations by the authors

***Means *p* < 0.001

The variance inflation factors (VIF) were tested to ensure that there was no multicollinearity between independent factors and dependent factors. Table 9 indicates that VIF values of all causal associations were not higher than 10, meaning that there was no problem of multicollinearity [80, 81]. The direct, indirect, and total effect values of each research path were further explored. As shown in Table 10, the largest impact on beneficiaries’ satisfaction was for EP (0.225), followed by SI (0.170), then BE (0.108), and PQ (0.085). Whereas the greatest impact on grid corporation trust, was recorded for PQ (0.333), followed by EP (0.246), then BS (0.182), BE (0.156), and SI (0.117). The indirect effect (0.128) of behavioral expectation on trust in State Grid was greater than its direct effect (0.029). The total effect value of behavioral expectation on environmental perception was 0.285, which was greater than the total effect value on BS (0.185), TSG (0.156), and PQ (0.143). It shows that behavior expectation affects the trust in State Grid more through environment perception and beneficiaries’ satisfaction, and the influence of environment perception on the trust in State Grid is greater.

**Table 10:** Direct effect, indirect effect and total effect value of each path

Hypothesized path	Direct effect value	Indirect effect value	Total effect value
BE < — SI	0.264	–	0.264
PQ < — SI	0.308	0.038	0.346
EP < — SI	− 0.340	0.162	− 0.178
BS < — SI	− 0.170	0.018	− 0.153
TSG < — SI	0.088	0.029	0.117
PQ < — BE	0.143	–	0.143
EP < — BE	0.245	0.040	0.285
BS < — BE	0.108	0.076	0.185
TSG < — BE	0.029	0.128	0.156
EP < — PQ	0.280	–	0.280
BS < — PQ	0.085	0.063	0.148
TSG < — PQ	0.248	0.084	0.333
BS < — EP	0.225	–	0.225
TSG < — EP	0.205	0.041	0.246
TSG < — BS	0.182	–	0.182

Source: The results based on the AMOS 23.0 software calculations by the authors

## Discussion

The results of the path coefficient analysis confirmed most of the proposed hypotheses. As shown in Table 9, the environmental perception was the most influential factor predicting beneficiaries’ satisfaction. This proves the importance of environmental perception in the residents’ satisfaction with PPAPs. Rural residents mostly rely on planting crops as their main source of income, while their incomes are affected by air or water pollution and extreme weather. Compared with traditional energy sources, the use of solar PV can greatly improve the current environmental situation. Therefore, rural residents are satisfied with PPAPs as they believed that solar PV can alleviate the current environmental problems. The previous study had also confirmed that there was a significant positive effect between passengers’ environmental perception and satisfaction [82]. The State Grid is involved in dealing with PV power generation and grid connection issues. Power supply stability can promote the extensive use of clean energy and reduce the use of traditional fossil fuels [83]. Therefore, as residents have a stronger perception of the environment, they can understand better that the efforts made by the State Grid will effectively solve environmental problems. Then, they are more likely to trust the State Grid. Some researches indicated that with higher individuals’ understanding of information literacy, more people will trust the website [84].

The social influence had a negative effect on beneficiaries’ satisfaction, while it had a positive effect on the trust in State Grid. The result is different from the previous researches [17, 50, 85]. Their research found that social influence had a greatly positive impact on users’ satisfaction. Users can know the products’ quality in advance through multiple channels of the network, so their satisfaction degree of the product was higher after rigorous screening and consideration. In this study, most beneficiaries are poverty-stricken households, whose understandings of the projects are mainly through the recommendation and publicity of village leaders, etc. There are gaps between the high expectation and the actual benefits of the projects, resulting in the lower satisfaction of residents with PPAPs. It is reasonable for the residents to have lower satisfaction with PPAPs in a short term, but this cannot prove that the residents won’t be perceived positive satisfaction in the long run.

According to the results, perceived quality was confirmed to have the strongest positive effect on the trust in State Grid and have a positive effect on beneficiaries’ satisfaction. As for the State Grid, its main responsibility is to ensure the efficient generation of PV panels. This will not only bring efficient use of household energy and cost savings to beneficiaries but also further improve the environmental quality. Therefore, the residents only perceive the reduction in household energy consumption and the improvement in environmental quality, and they will believe that the State Grid has fulfilled its responsibilities. This is similar to the results by Sarkar and Chen [36, 71]. Furthermore, residents' satisfaction with PPAPs increases when they realize the benefits that adopting solar PV can bring to their families and communities. In previous researches, the users’ perception of solar home systems’ benefits and the reduction in their energy costs had a critical impact on their satisfaction [27].

Behavioral expectation can directly facilitate beneficiaries’ satisfaction but indirectly encourage their trust in State Grid. At present, PPAPs still rely on government publicity and support. Therefore, if the government can promulgate policies, subsidies, and other support for solar PV in time, the residents’ satisfaction with PPAPs will be enhanced, which was similar to the results found by Zhang and Shen [54, 57]. In this paper, behavioral expectation refers to the residents’ expectation of the government’s support on PPAPs, so it may not have a direct significant impact on their trust in State Grid. However, when the residents realize the benefits of PPAPs in their lives, such as reduction in household energy expenditure, they may believe that the State Grid plays a vital role in PPAPs. In this way, their trust in State Grid can be enhanced. Therefore, even though behavioral expectation had no direct effect on the trust in State Grid, it can be an essential factor for the trust in State Grid through perceived quality. Likewise, customer expectation was confirmed to have an indirect role in promoting social trust [86].

The results of this study supported the hypothesis that beneficiaries’ satisfaction had a positive impact on the trust in State Grid. This indicates that the more satisfied the residents are with PPAPs, the more they will trust in State Grid. China State Grid implements a “one-stop service” to ensure safe and stable operations of PV equipment, whether it is in the early stage, mid-stream maintenance, or later tracking services, etc. However, these all involve the relationship between beneficiaries and the State Grid. When the residents are satisfied with PPAPs, meaning that they are satisfied with the services provided by the State Grid, then they will rely on State Grid and continue to participate in PPAPs. The predecessors also found a significant role between trust and customers’ satisfaction [70, 87]. Inadequate power supply and unreliable power service will lead to end-users dissatisfaction with power service [88].

### Theoretical implications

As discussed in the literature review, few scholars have studied the direct relationship between satisfaction and trust from the perspective of beneficiaries. In addition, most satisfaction models were based on ACSI [16] to test customers’ satisfaction or explore the relationship between satisfaction and users’ complaints [30], loyalty [89], or adoption willingness [17].

This study, hence, establishes an integrated model based on ACSI and UTAUT with the dimension of “environmental perception” to examine the beneficiary perception about the environmental benefits of PPAPs and provides a new dimension and theoretical models for critical aspects that beneficiaries should consider in the process of building PPAPs’ satisfaction.

### Limitations and future research directions

Although this study has enriched our understandings of the current implementation of PPAPs in China, some limitations still exist. First, the data used in this paper are cross-sectional, since the implementation period of PPAP in China is not long. The data can reflect the current views of the beneficiaries on the projects, but it cannot show the changed satisfaction of the beneficiaries in the process of PPAPs. Therefore, in future researches, longitudinal research is needed to find out the factors that affect the PPAPs’ beneficiaries’ satisfaction over time. Second, although the current research model covers many factors, other factors involving household energy usage, rural residents’ cognition, and power supply stability before and after project implementation, etc. can also be considered in future researches to fully explain the reasons for affecting the sustainable development of PPAPs. In addition, this study has not considered the impact of family cultural factors (such as energy-saving habits, family size, and lifestyle). Future studies will ponder over these cultural aspects to enrich current understandings of the main factors that hinder or support the success of PPAPs.

## Conclusions and policy implications

### Conclusions

This study attempts to provide more understandings about what may affect beneficiaries’ satisfaction with PPAPs and their trust in State Grid. Behavioral expectation (BE) and perceived quality (PQ) was used to predict beneficiaries’ satisfaction in this paper. Environmental Perception (EP) was also considered as the unique feature of PPAPs. The following conclusions are drawn:

1. *Environmental perception was the most influential factor predicting beneficiaries’ satisfaction*. It means that when residents’ environmental perception is enhanced, they will be more satisfied with PPAPs.
2. *Social influence had a negative impact on beneficiaries’ satisfaction*. Social influence means the government officials and village leaders who persuade beneficiaries to adopt solar PV. If the government or village leaders excessively force the residents to participate in the PPAPs, it may make the residents feel resistant instead.
3. *Perceived quality had the most important impact on the trust in State Grid*. The State Grid plays an important role in the perception of quality changes for residents in family energy use and environmental problems after adopting solar PV. Perceiving the benefits of adopting renewable energy, they are more likely to trust the State Grid.
4. *Behavioral expectation had an indirect impact on the trust in State Grid*. Behavior expectation is more likely the beneficiaries expected the government’s behavior with PPAPs, while the State Grid will take a series of measures according to the government’s policies to produce indirect effects.

Through the exploration of these factors, it may be of reference significance for other developing countries to carry out renewable energy poverty alleviation projects. First of all, the developing countries should pay attention to raising residents’ environmental awareness and popularizing the advantages of renewable energy in energy conservation and environmental protection. Second, the influence of the government and village leaders in the propaganda process should not be underestimated. Finally, in the implementation of renewable energy projects, the State Grid plays an important role and establishing a good relationship of trust with the residents can improve residents’ satisfaction with the project.

### Policy implications

Based on the above further practical and empirical understanding of the main factors that should be considered for the sustainable development of solar PV, this study proposes the following policy implications from the behavior perspective of the government, village leaders, and the State Grid:

1. The government and village leaders need to explain the current environmental problems to residents for boosting the use of renewable energy and make concerted the greatest efforts to help residents better understand that solar PV can bring more environmental benefits compared with traditional energy (including coal, fuelwood, straw, etc.). The State Grid should also promptly explain the current implementation of renewable energy projects to residents. By doing this, residents can understand the improvement of environmental problems because of these projects. Only by enhancing the environmental perception of the residents, can their satisfaction and trust in State Grid be increased.
2. The government or village leaders should enhance residents’ understanding of PPAPs by conducting appropriate publicity and training. In addition, through sharing their personal experience of using PPAPs and recommending suitable PPAPs’ type to residents. When promoting the implementation of PPAPs, the government or village leaders should also first let the residents fully understand the credibility of the State Grid in PPAPs, to increase the trust in State Grid.
3. To enhance the residents’ quality perception of household energy usage and expenditure, they should first explain the operation, maintenance, service, and quality assurance of PPAPs throughout the process to residents. At the same time, to ensure the reliability of the data obtained by the beneficiaries and the professional quality of the equipment, the State Grid’s technicians should implement full-tracking services and regularly maintain the equipment in PPAPs, thus the residents can learn more about the transformation of the quality of household energy. The low quality of equipment and high energy cost both have a negative impact on the satisfaction of households using solar PV [27]. Therefore, learning about the integration of the PPAPs’ implementation process can improve residents’ perceived quality.
4. The residents expect the government’s subsidies, policies, and maintenance of facilities. Affected by the epidemic COVID-19, China has made appropriate adjustments to the distribution of PV poverty alleviation benefits and electricity prices. These policy changes should be greatly publicized by the local government and village leaders. And then, residents can perceive the government’s full support for PPAPs. Accordingly, the State Grid should also adjust electricity prices promptly so that residents can perceive improvements in household energy quality, promoting their trust in the State Grid in an underlying manner.

## Data Availability

Not applicable.

## Appendix

See Fig. 4 and Table 11.

**Table 11:** Abbreviations

Abbr.	Full name
RMR	Root Mean square Residual
SRMR	Standardized Root Mean square Residual
RMSEA	Root Mean Square Error of Approximation
GFI	Goodness of Fit Index
AGFI	Adjusted Goodness of Fit Index
NFI	Normed Fit Index
RFI	Relative Fit Index
IFI	Incremental Fit Index
TLI	Tucker–Lewis Index
CFI	Comparative Fit Index
PGFI	Parsimony Goodness of Fit Index
PNFI	Parsimony Normed Fit Index
CN	Critical N
PCFI	Parsimony Comparative Index
